# Incidence and risk factors for postoperative nausea and vomiting following video-assisted thoracoscopic surgery: a prospective observational study

**DOI:** 10.3389/fmed.2025.1714897

**Published:** 2026-03-10

**Authors:** Jing Zhang, Jing Ma, Liang Jin, Yi Liu, Xueyao Yu, Jinjin Huo, Ning Kang, Shuang Gao, Yuanhang Zhang, Liyun Bao, Wei Liu, Liyuan Hao, Li Fan, Jiechu Wang, Xiangyang Guo, Ning Yang

**Affiliations:** 1 Department of Anesthesiology, Peking University Third Hospital, Beijing, China; 2 Department of Thoracic Surgery, Peking University Third Hospital, Beijing, China; 3 Department of Laboratory Medicine, Peking University Third Hospital, Beijing, China

**Keywords:** general anesthesia, postoperative nausea and vomiting (PONV), prospective study, risk factors, video-assisted thoracoscopic lung surgery (VATS)

## Abstract

**Background:**

Postoperative nausea and vomiting (PONV) is a common complication after general anesthesia, contributing to patient discomfort, adverse clinical outcomes, and increased healthcare costs. While video-assisted thoracoscopic surgery (VATS) offers substantial benefits over open procedures, data on the incidence and predictors of PONV specifically following VATS are scarce and predominantly derived from retrospective studies. This study aimed to prospectively determine the incidence and identify independent risk factors for PONV within the first 72 h after VATS.

**Methods:**

In this single-center, prospective observational study, we enrolled 355 adult patients (aged ≥ 18 years) who underwent VATS under general anesthesia at Peking University Third Hospital between September 2022 and September 2023. PONV was assessed in the post-anesthesia care unit (PACU) within 2 h postoperatively and then daily on the ward for three consecutive days. The primary outcome was the incidence of PONV within 72 h. Multivariate logistic regression was employed to identify independent risk factors, with a *P*-value < 0.05 deemed statistically significant.

**Results:**

The overall incidence of PONV within 72 h was 19.44% (69/355). PONV was significantly more frequent in female patients (84.06% of PONV cases) than in males (15.94%, *P* < 0.001). The highest incidence occurred in the PACU (17.96%), followed by the day of surgery (7.89%) and postoperative day 1 (POD1, 7.61%) on the ward. The incidence decreased to 0.85% on POD2 and 1.41% on POD3. The most severe PONV episodes were reported on the day of surgery (17.86% of PONV cases) and on POD1 (22.22%). Multivariate analysis identified four independent risk factors: female gender, preoperative erector spinae plane block (ESPB), non-administration of preoperative steroids, and low intraoperative minimum mean arterial pressure (MAP).

**Conclusion:**

Approximately one in five patients experienced PONV within 72 h after VATS. Independent risk factors included female gender, preoperative ESPB, omission of preoperative steroid prophylaxis, and low intraoperative minimum MAP. These findings highlight potential targets for risk stratification and optimized PONV prophylaxis in this surgical population.

## Introduction

Postoperative nausea and vomiting (PONV) is a frequent and undesirable complication following surgical procedures. Reported incidence rates approximate 30% across general surgical populations, but can escalate to as high as 80% among high-risk cohorts (1). PONV can precipitate a range of adverse sequelae, including wound dehiscence, hemorrhage, dehydration, electrolyte disturbances, and aspiration pneumonia. These complications contribute to significant patient discomfort, extended hospitalization, and elevated healthcare expenditures (1–4). Despite the implementation of diverse prophylactic and therapeutic strategies by anesthesiologists, the incidence of PONV persists at 15–30% (2, 5, 6). Therefore, PONV is consistently considered as a core quality metric in perioperative care (7). This underscores a pressing need to identify more precise predictive factors and enhance preventative protocols for PONV. Extant research on PONV risk factors has predominantly concentrated on surgical types associated with high incidence, such as abdominal, gynecological endoscopic, ophthalmic strabismus, otolaryngological, oral, and orthopedic surgeries. In contrast, investigations into PONV following thoracic surgery remain relatively scarce. Lung cancer, the second most commonly diagnosed malignancy globally, was the leading cause of cancer-related mortality in 2020 (8). Surgical resection constitutes the cornerstone of treatment for early-stage disease. Video-assisted thoracoscopic surgery (VATS) has emerged as a minimally invasive alternative to open thoracotomy for lobectomy in early-stage non-small cell lung cancer, offering significant clinical benefits including reduced postoperative pain and improved quality of life (9). However, PONV following VATS is a notable concern, adversely affecting patient comfort and potentially leading to serious complications such as dehydration, electrolyte imbalances, venous hypertension, esophageal rupture, wound suture separation, and life-threatening airway obstruction from aspiration (10). To date, few studies have specifically investigated the etiology of PONV in VATS patients. Existing literature primarily associates it with postoperative analgesic regimens (11–14) or is derived from retrospective analyses (10, 15–19) (20). Therefore, the primary objective of this prospective observational study is to determine the incidence and identify the independent risk factors for PONV during the first 72 h following VATS performed under general anesthesia. The findings aim to inform targeted strategies for mitigating PONV in this surgical population, thereby facilitating enhanced recovery.

## Materials and methods

### Trial design and ethics

This prospective, single-center, observational study enrolled patients scheduled for VATS at Peking University Third Hospital, China, between September 2022 and September 2023. The study was conducted in accordance with the ethical principles of the Declaration of Helsinki (2013) and received approval from the Medical Science Research Ethics Committee of Peking University Third Hospital (IRB00006761-M2022575). The trial was registered with the Chinese Clinical Trial Registry (ChiCTR2200066231). Written informed consent was obtained from all participants prior to their inclusion in the study.

### Participants

Eligible participants were adults (age ≥ 18 years) admitted for VATS, with an American Society of Anesthesiologists (ASA) physical status of I to III. Exclusion criteria comprised: receipt of preoperative radiotherapy or chemotherapy; severe cardiovascular or cerebrovascular disease; severe hepatic or renal dysfunction; inability to cooperate (e.g., due to language barriers or mental illness); unstable angina or myocardial infarction within the preceding 3 months; ASA grade > III; scheduled for a second thoracic surgery; unplanned secondary thoracotomy; postoperative transfer to the intensive care unit (ICU); and unwillingness to participate or unexpected early discharge.

### Preoperative baseline data collection and evaluation

Baseline data were collected during preoperative interviews conducted by trained investigators on the day before surgery. Data included demographic characteristics, laboratory results, ASA status, specific surgical type, comorbidities, past medical history, and history of PONV. Participants retained the right to withdraw from the study at any time without providing a reason and without prejudice to their standard medical care.

### Anesthesia and analgesia protocal

A standardized anesthetic protocol was administered to all participants. Standard monitoring included electrocardiography, pulse oximetry, capnography, non-invasive blood pressure measurement, and bispectral index (BIS). Prior to induction, in the lateral decubitus position, patients received an ultrasound-guided erector spinae plane block (ESPB) at the T5 level on the operative side, performed by a single anesthesiologist, as previously described (21). Briefly, using a high-frequency linear ultrasound probe, 20 mL of 0.375% ropivacaine was injected deep to the erector spinae muscle after confirming correct needle placement. Spread of local anesthetic was confirmed from T3 to T7.

Anesthesia induction was standardized with intravenous sufentanil (0.3–0.5 μg/kg), etomidate (0.2–0.3 mg/kg), and propofol (1.5–2.0 mg/kg), facilitated by cisatracurium (0.15–0.2 mg/kg). Maintenance was achieved using a target-controlled infusion of propofol to maintain a Bispectral Index (BIS) of 40–60, combined with remifentanil infusion (0.1–0.2 μg/kg/min). Dexamethasone (5 mg) was administered immediately post-induction, while ondansetron (4 mg) was administered 30 min prior to the end of surgery. Ventilation was set at a tidal volume of 6–8 mL/kg ideal body weight during double-lung ventilation and 4–6 mL/kg during one-lung ventilation, with an inspiratory-to-expiratory ratio of 1:2. End-tidal carbon dioxide (PetCO2) was maintained at 35–45 mmHg (1 mmHg = 0.133 kPa), and oxygen saturation above 95%. Anesthesia was maintained with sevoflurane, propofol, and remifentanil, titrated to maintain a BIS value between 40 and 60. The use of fentanyl and midazolam was prohibited. Intraoperative hypotension (defined as a > 20% decrease from baseline or MAP < 65 mmHg) was managed with norepinephrine, and bradycardia (heart rate < 50 beats per minute) with atropine (6, 21).

All surgeries were performed by the same surgical team via VATS in the lateral decubitus position. A chest tube was placed postoperatively. Postoperative analgesia consisted of an intercostal nerve block administered by the surgeon, supplemented with patient-controlled intravenous analgesia (PCIA) containing sufentanil (100 μg) and ondansetron (8 mg) in 100 mL saline, continued for 48 h. Rescue analgesia with intravenous parecoxib sodium (40 mg) was provided for inadequate pain control (VAS ≥ 4). Prophylactic antiemetics (ondansetron 8 mg or metoclopramide 20 mg) were administered at the end of surgery. Rescue antiemetics were given for vomiting or persistent nausea lasting ≥ 2 h. Patients were extubated and transferred to the post-anesthesia care unit (PACU), with discharge from PACU with an Aldrete score ≥ 8 (22). Intraoperative data, including anesthesia/surgery durations, hemodynamic parameters, and drug doses, were recorded. If a patient developed complications during anesthesia, such as hypotension or low oxygen saturation, these were recorded accordingly, and anesthesiologists administered treatment in accordance with clinical recommendations.

### Postoperative assessment

Trained investigators, blinded to group assignment, conducted assessments at fixed intervals: immediately upon arrival in the PACU, at 2 h (prior to ward transfer), and subsequently daily at 24, 48, and 72 h postoperatively. Episodes were recorded based on both patient self-reporting and nursing charts. Assessments included the incidence of PONV, use of rescue antiemetics, and PONV severity.

### Outcomes

The primary outcome was the incidence of PONV within 72 h after surgery. PONV was defined as the occurrence of nausea (subjective urge to vomit), retching (unproductive vomiting efforts), or vomiting (forceful expulsion of gastric contents).

Secondary outcomes included: (1) Severity of PONV (0–72 h), assessed using a 4-point Likert scale (0 = none, 1 = mild, 2 = moderate, 3 = severe) (23); (2) Use of rescue antiemetics (0–72 h); (3) Requirement for supplementary analgesics (0–72 h); (4) Anesthesia time; (5) Surgical time; (6) Incidence of adverse events, specifically defined as post-induction hypotension, intraoperative hypotension, intraoperative hypertension, intraoperative bradycardia, and intraoperative tachycardia; (7) Length of hospital stay (from the day of surgery to discharge); (8) Total hospitalization costs (in RMB, post-insurance).

### Grouping method

Patients were categorized into two groups based on the occurrence of PONV within 72 h: the Non-PONV group and the PONV group. Chi-square and non-parametric tests were performed on variables including gender, age, BMI, ASA grade, underlying diseases, smoking history, motion sickness history, nerve block mode, analgesia methods, steroid usage, intraoperative PONV prophylaxis, intraoperative fluid rehydration amount, anesthesia time, operation time, and surgical method. Variables with *P* < 0.20 in Chi-square and non-parametric tests were included in the multivariate logistic regression analysis.

### Sample size calculation

The sample size calculation was predicated on a baseline PONV incidence of approximately 30% in this surgical population (24, 25), as indicated by previous literature. To detect a clinically significant reduction or variance with a power of 80% and an alpha error of 0.05, considering 10 potential predictor variables in a logistic regression model (requiring ∼10 events per variable) (24, 26, 27), we estimated a minimum sample size of 330. Adjusting for a 10% attrition rate, the target enrollment was set at 360 patients.

### Statistical analysis

All statistical analyses were performed using Statistical Package for Social Sciences (SPSS) version 22.0 (IBM Corp., Armonk, NY, United States). Non-normally distributed continuous variables were represented by the median. Categorical variables were expressed as number (%). Chi-square and non-parametric tests were performed to analyze demographic data, past medical history, as well as baseline and intraoperative data. Multicollinearity among independent variables was assessed using the Variance Inflation Factor (VIF), with a threshold of < 5 considered acceptable. Variables with *P* < 0.20 in univariate analysis were entered into a multivariate logistic regression model using a backward stepwise elimination method. All tests were two-sided, and *P* < 0.05 was considered statistically significant.

## Results

### Study flow chart

**FIGURE 1:**
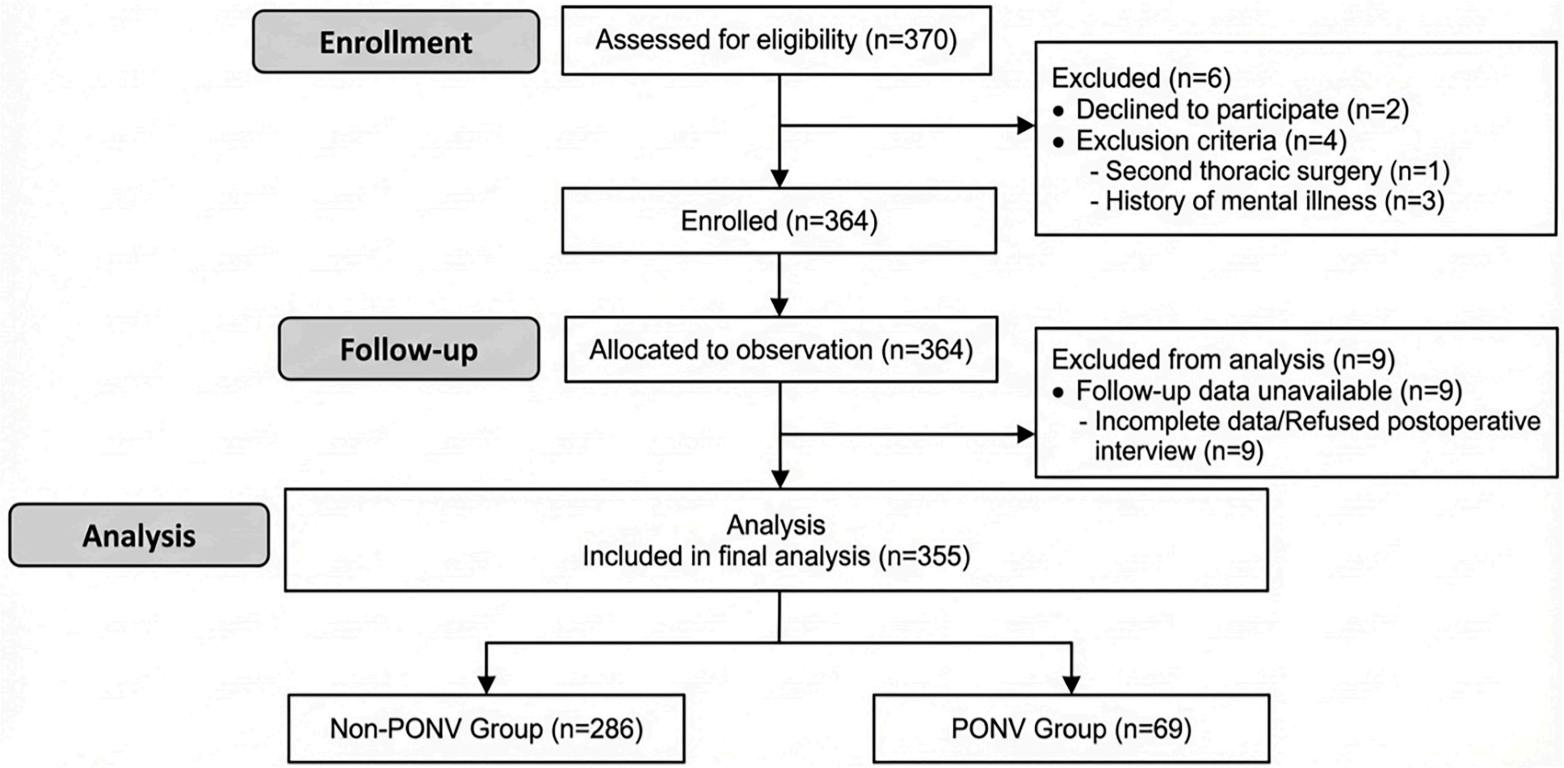
Flow chart of the trial. ASA, American Society of Anesthesiologists.

A total of 370 adult patients were initially screened for eligibility. Of these, 364 met the inclusion criteria and were enrolled. After exclusions, 355 patients were included in the final data analysis (Figure 1).

### The outcomes

The overall incidence of PONV within 72 h after surgery was 19.44% (69/355). Among the patients who experienced PONV, 84.06% (58/69) were female and 15.94% (11/69) were male. The incidence of PONV in the post-anesthesia care unit (PACU) was 17.96% (44/245). On the day of surgery and postoperative day 1, the incidence rates in the ward were 7.89% (28/355) and 7.61% (27/355), respectively. The incidence decreased to 0.85% (3/355) on postoperative day 2 and 1.41% (5/355) on postoperative day 3 (Table 1). The highest proportion of severe PONV (Likert scale score of 3) was observed on the day of surgery (17.86%, 5/28) and on postoperative day 1 (22.22%, 6/27) among those who experienced PONV at those time points (Table 2).

**TABLE 1:** The temporal patterns and incidence of PONV in patients undergoing VATS.

Time	Numbers	Incidence (*n* = 355, %)
PACU (*n* = 245)	44	17.96
In the ward on the day of surgery (*n* = 355)	28	7.89
Postoperative day 1 (*n* = 355)	27	7.61
Postoperative day 2 (*n* = 355)	3	0.85
Postoperative day 3 (*n* = 355)	5	1.41

Data are presented as number. PONV, postoperative nausea and vomiting; VATS, video-assisted thoracoscopic surgery; PACU, post-anesthesia care unit.

**TABLE 2:** The severity of PONV in patients within 72 h after VATS.

Severity of PONV, No.	PACU (*n* = 245)	In the ward on the day of surgery (*n* = 355)	Postoperative day 1 (*n* = 355)	Postoperative day 2 (*n* = 355)	Postoperative day 3 (*n* = 355)
0	201	327	328	352	350
1	39	20	18	3	5
2	4	3	3	0	0
3	1	5	6	0	0
Total	44	28	27	3	5

Data are presented as number. PONV, postoperative nausea and vomiting; VATS, video-assisted thoracoscopic surgery; PACU, post-anesthesia care unit.

### Comparison of baseline characteristics

Baseline characteristics were compared between the Non-PONV and PONV groups using Chi-square and non-parametric tests. No statistically significant differences were observed in age, BMI, years of education, preoperative anxiety score, history of motion sickness or PONV, smoking history, diabetes mellitus, preoperative platelet count (PLT), alanine aminotransferase (ALT), aspartate aminotransferase (AST), albumin (ALB), blood glucose (Glu) levels, baseline diastolic blood pressure, or baseline mean arterial pressure (MAP) (all *P* > 0.05). Significant differences were found between the two groups in gender, history of cardiovascular diseases, preoperative white blood cell count (WBC), red blood cell count (RBC), hemoglobin concentration (HGB), hematocrit (HCT), creatinine level, and baseline systolic blood pressure (all *P* < 0.05), suggesting an association between these variables and the occurrence of PONV (Table 3).

**TABLE 3:** Comparison of the preoperative data between Non-PONV and PONV groups.

Variable	Non-PONV group (*n* = 286)	PONV group (*n* = 69)	*P*-value
Age, median (IQR), yr	57(43.75–64.25)	53(47–65)	0.895
Female (n,%)	161(56.3)	58(84.1)	<0.001
BMI, median (IQR), kg/m^2^	24.31(22.03–26.86)	23.59(21.03–25.22)	0.064
Duration of education, median (IQR), yr	12(9–16)	15(–916)	0.501
Preoperative anxiety score, Median (IQR)	3(0–5)	3(3–6)	0.247
Motion sickness or history of PONV, No. (%)	22(7.7)	10(14.5)	0.077
Smoking, No. (%)	59(20.6)	10(14.5)	0.248
Cardiovascular diseases, No. (%)	110(38.5)	17(25.0)	0.038
Diabetes mellitus, No. (%)	31(10.8)	4(5.8)	0.207
WBC values on preoperative day 1, Median (IQR), *10^9^/L	5.82(4.98–7.00)	5.31(4.–546.36)	0.004
RBC values on preoperative day 1, Median (IQR), *10^12^/L	4.58(4.33–4.94)	4.52(4.18–4.76)	0.027
HGB values on preoperative day 1, Median (IQR), g/L	138(129–150)	134(124.5–145)	0.012
PLT values on preoperative day 1, Median (IQR), *10^9^/L	227(193.8–271.3)	209(189–258.5)	0.228
HCT values on preoperative day 1, Median (IQR)	0.42(0.40–0.45)	0.41(0.38–0.44)	0.013
ALT values on preoperative day 1, Median (IQR), U/L	18(13.75–26.25)	18(13–28)	0.927
AST values on preoperative day 1, Median (IQR), U/L	22(18–26)	22(19–26)	0.917
Cr values on preoperative day 1, Median (IQR), μmol/L	73.5(65.0–86.3)	68(60.5–74)	<0.001
ALB values on preoperative day 1, Median (IQR), g/L	45.6(43.65–47.4)	44.9(43.4–47.25)	0.518
Glu values on preoperative day 1, Median (IQR), mmol/L	5.5(5.0–6.1)	5.4(4.9–5.9)	0.279
Baseline systolic blood pressure, Median (IQR), mmHg	128(120–139)	123.5(118.5–133.5)	0.039
Baseline diastolic blood pressure, Median (IQR), mmHg	78(70–81)	78(70–82)	0.617
Baseline mean arterial pressure (MAP), Median (IQR), mmHg	93(87–101.25)	92(86–99)	0.136

Data are presented as median (IQR) or number (%). IQR, inter-quartile range; PONV, postoperative nausea and vomiting; BMI, body mass index; WBC, white blood cell; RBC, red blood cell; HGB, hemoglobin; PLT, platelet; HCT, hematocrit; ALT, alanine.

### Comparison of intraoperative data

No significant differences were found between the groups in terms of maximum and minimum systolic and diastolic blood pressures, duration of intraoperative hypotension or hypertension, intraoperative prophylactic antiemetic use, anesthesia technique (total intravenous, total inhalation, or combined anesthesia), total sufentanil dosage, use of patient-controlled intravenous analgesia (PCIA), use of erector spinae plane block (ESPB), use of tramadol, use of dexmedetomidine, anesthesia time, or operation time (all *P* > 0.05). However, preoperative steroid use and intraoperative minimum MAP were significantly associated with PONV occurrence (both *P* < 0.05) (Table 4).

**TABLE 4:** Comparison of the intraoperative data between Non-PONV and PONV groups.

Variable	Non-PONV group (*n* = 286)	PONV group (*n* = 69)	*P*-value
Preoperative use of steroid drugs, No. (%)	187(65.4)	35(50.7)	0.024
The maximal systolic blood pressure, Median (IQR), mmHg	134(123–148)	140(118.5–149.75)	0.692
The maximal diastolic blood pressure, Median (IQR), mmHg	79(70–89)	80(70–88)	0.827
The minimal systolic blood pressure, median (IQR), mmHg	96(90–104)	93(86.25–102.75)	0.333
The minimal diastolic blood pressure, Median (IQR), mmHg	56.5(50–64.25)	57(51–64.5)	0.953
The minimal mean arterial pressure (MAP), Median (IQR), mmHg	70(63–77)	66(57–76)	0.002
The duration of intraoperative hypotension, Median (IQR), min	1(0–5)	1(0–5)	0.684
The duration of intraoperative hypertension, Median (IQR), min	0(0–1)	0(0–1	0.608
Intraoperative prophylactic medication for PONV, No. (%)	262(91.6)	59(86.8)	0.217
Anesthesia method, No. (%)			0.700
Intravenous anesthesia alone	102(35.7)	22(32.4)	
Inhalation anesthesia alone	131(45.8)	27(39.7)	
Combined anesthesia	53(18.5)	19(27.9)	
Total intraoperative sufentanil dosage, Median (IQR), μg	115(115–120)	112(110–120)	0.438
Total intraoperative fluid volume, Median (IQR), mL	1,100(1,100–1,550)	1,100(1,000–1,100)	0.056
Tramadol, No. (%)	16(5.6)	5(7.2)	0.602
Dexmedetomidine, No. (%)	16(5.6)	3(4.3)	0.680
PCIA, No. (%)	230(80.4)	55(79.7)	0.894
Erector spinae plane block (ESPB), No. (%)	10(3.5)	6(8.7)	0.062
Anesthesia time, Median (IQR), min	153(120–180)	144(110–178)	0.216
Operation time, Median (IQR), min	103(77–128.5)	100(74–131)	0.606
Length of postoperative hospital stay, Median (IQR), days	3(3–4)	3(3–4)	0.128
Total hospitalization costs, Median (IQR), RMB	56376.7(42460.87–73822.25)	52600.65(45077.62-67391.77)	0.704

Data are presented as median (IQR) or number (%). IQR, inter-quartile range; PONV, postoperative nausea and vomiting; PCIA, patient-controlled intravenous analgesia; RMB, renminbi.

### Multivariate logistic regression analysis of risk factors for PONV

Variables with *P* < 0.20 in chi-square and non-parametric tests were analyzed by multivariate logistic regression. Multivariate logistic regression analysis identified four independent risk factors for PONV: female gender (OR 2.919; 95%CI 1.086–7.845; *P* = 0.034), preoperative erector spinae plane block (ESPB) (OR 3.350; 95%CI 1.032–10.880; *P* = 0.044), preoperative non-use of steroid drugs (OR 0.496; 95%CI 0.274–0.897; *P* = 0.020), and low intraoperative minimal MAP (OR 0.967; 95%CI 0.937–0.998; *P* = 0.035) (Table 5). Furthermore, *post hoc* interaction analyses were performed to evaluate potential synergistic effects between key variables, specifically Gender × Steroid use and ESPB × MAP. No statistically significant interactions were observed (*P* > 0.05 for all terms), suggesting that these risk factors exert their influence independently.

**TABLE 5:** Multivariate logistic regression analysis of risk factors of PONV in patients undergoing VATS.

Risk factors	OR	95%CI	*P*-value
Gender	2.919	1.086–7.845	0.034
Motion sickness or history of PONV	1.230	0.490–3.084	0.660
Cardiovascular diseases	0.680	0.334–1.385	0.288
HGB values on preoperative day 1	1.005	0.986–1.024	0.641
Cr values on preoperative day 1	0.995	0.968–1.023	0.728
Baseline systolic blood pressure	0.991	0.968–1.013	0.414
Erector spinae plane block (ESPB)	3.350	1.032–10.880	0.044
Preoperative use of steroid drugs	0.496	0.274–0.897	0.020
The minimal intraoperative mean arterial pressure (MAP)	0.967	0.937–0.998	0.035
Total intraoperative fluid volume	0.999	0.998–1.000	0.179

Data are presented as number. IQR, inter-quartile range; PONV, postoperative nausea and vomiting; HGB, hemoglobin; Cr, creatinine.

## Discussion

In this prospective single-center observational study, we investigated the incidence and risk factors of PONV within the first 72 h following VATS under general anesthesia. The overall incidence of PONV was 19.44%. This incidence rate aligns with the predicted probability (approximately 20%) for patients possessing two Apfel risk factors (e.g., female gender and non-smoking status), lending external validity to our cohort despite the lack of prospective Apfel stratification. The highest incidence was observed in the PACU (17.96%), with a progressive decline to 0.85% by POD 2 and 1.41% by POD 3. This pattern underscores the transient nature of PONV and is consistent with previous studies highlighting the early postoperative period as a high-risk window attributable to residual anesthetic effects and opioid administration (1, 6, 28, 29). The low incidence of late-onset PONV may reflect effective prophylactic strategies, as 91.6% of patients without PONV received antiemetic prophylaxis. Notably, PONV severity was predominantly mild (Grade 1) throughout the 72-h period, suggesting that although most cases were not severe, proactive management remains necessary to prevent clinical deterioration (28, 30). Multivariate analysis identified several factors significantly associated with PONV, including female gender, preoperative erector spinae plane block (ESPB), non-use of preoperative steroids, and lower intraoperative minimum MAP. These findings are largely consistent with established risk models while also providing novel insights specific to the VATS population.

Female gender was a strong independent predictor of PONV, with an adjusted odds ratio (OR) of 2.919 (*P* = 0.034). Among patients who experienced PONV, 84.1% were female. This result aligns with extensive evidence indicating that female gender is among the strongest risk factors for PONV, associated with a two- to four-fold increase in incidence compared to males (31, 32). The pathophysiology underlying this gender disparity remains incompletely elucidated. Current evidence suggests that nausea involves forebrain pathways, while the vomiting center is located in the medulla oblongata (33). The emetic reflex is a complex process initiated by chemical stimuli that trigger the release of neurotransmitters such as serotonin and substance P, activating visceral vagal afferents and ultimately stimulating the dorsal vagal complex in the brainstem (33, 34). Some studies propose that cyclical variations in female hormone levels may increase serotonin concentrations and enhance chemoreceptor sensitivity to emetogenic stimuli (35, 36). However, Apfel et al. reported no association between menstrual cycle phase or menopausal status and PONV risk (24), indicating that the exact mechanisms require further investigation.

A lower minimum intraoperative MAP was also associated with an elevated PONV risk (OR = 0.967, *P* = 0.035). This observation is consistent with previous reports linking intraoperative hypotension to a higher incidence of PONV (37, 38). A history of preoperative hypotension or orthostatic intolerance has also been identified as a risk factor (39). The mechanism may involve reduced cerebral perfusion affecting brainstem regions involved in emesis, such as the chemoreceptor trigger zone (37–40).

Contrary to expectations and previous literature suggesting that regional analgesia techniques reduce opioid consumption and PONV incidence (41–44), preoperative ESPB was associated with an increased risk of PONV in our cohort (OR = 3.350, 95% CI: 1.032–10.880, *P* = 0.044). The association between preoperative ESPB and increased PONV risk presents a seeming paradox, given the opioid-sparing potential of regional anesthesia. However, we postulate that the extensive sympathetic blockade induced by ESPB may precipitate intraoperative hypotension, a known independent predictor of PONV. Reduced perfusion to the chemoreceptor trigger zone and gut mucosal ischemia may lower the emetic threshold, potentially offsetting the benefits of reduced opioid consumption. Furthermore, the localized spread of local anesthetics might exert subtle systemic effects that warrant further pharmacokinetic investigation. It is also crucial to note that our anesthetic protocol involved the use of sufentanil. The persistence of PONV suggests that regional techniques like ESPB may not fully mitigate risk if the emetogenic threshold is crossed by systemic opioids. This highlights the potential necessity of a strictly opioid-free anesthesia (OFA) strategy to fully realize the antiemetic benefits of regional blockade in thoracic surgery.

Preoperative administration of corticosteroids has been well-established to reduce PONV incidence (45, 46). A meta-analysis indicated comparable efficacy between 4-5 mg and 10 mg dexamethasone doses (45). and its prophylactic effect is similar to that of 5-HT_3_ receptor antagonists such as ondansetron (47). In this study, 5 mg dexamethasone was administered prior to induction. The protective effect of corticosteroids is thought to involve anti-inflammatory mechanisms and modulation of central neurotransmitter activity (48).

In contrast, lower preoperative white blood cell and hemoglobin levels, though significant in univariate analysis, were not independent predictors in the multivariate model, suggesting they may serve as surrogate markers rather than direct causal factors. Similarly, although preoperative creatinine levels differed between groups (*P* < 0.001), their clinical relevance to PONV remains uncertain. Our identified risk profile offers a nuanced counterpoint to the established Apfel simplified risk score (31). While we corroborated the robust predictive value of female gender, a cornerstone of the Apfel model, other components behaved differently in this thoracic cohort. Notably, a history of motion sickness or prior PONV did not retain statistical significance in our multivariate analysis (*P* = 0.077); this divergence may be attributable to the uniform administration of dual antiemetic prophylaxis (dexamethasone and ondansetron), which likely attenuated the clinical expression of this baseline susceptibility. Furthermore, whereas the Apfel score emphasizes postoperative opioid use, our findings suggest that in the context of VATS, intraoperative hemodynamic parameters—specifically low minimum MAP—and the choice of regional blockade (ESPB) act as critical, procedure-specific determinants. This implies that generic risk stratification tools may require refinement to incorporate hemodynamic variables for optimal precision in thoracic surgery.

Several limitations of this study should be acknowledged. First, the single-center design may limit generalizability. Future multi-center randomized controlled trials including more diverse patient populations are warranted. Second, we did not perform preoperative Apfel risk scoring, measure perioperative inflammatory or other PONV-related biomarkers, or stratify procedures by surgical subtype. The multifactorial nature of PONV implies that numerous unmeasured confounders may have influenced our results, which may explain why several factors significant in univariate analysis did not retain significance in the multivariate model. Third, while we adjusted for surgical duration, we did not stratify outcomes by specific procedure type (e.g., lobectomy versus wedge resection) due to the predominance of lobectomies in our cohort. Future studies with larger sample sizes should investigate whether the extent of pulmonary resection differentially impacts PONV risk. Therefore, further well-designed studies are needed to validate and extend our findings.

## Conclusion

This study establishes that female sex, preoperative ESPB, omission of preoperative steroids and intraoperative hypotension independently predict PONV after VATS. These results underscore the need for targeted multimodal prophylaxis (e.g., dexamethasone and 5-HT3 antagonists) in high-risk groups, especially women and hypotensive patients. The analgesic advantages of regional techniques like ESPB must be balanced against their PONV risk. Enhanced PACU monitoring is also essential to detect the characteristic early onset of symptoms.

## Data Availability

The raw data supporting the conclusions of this article will be made available by the authors, without undue reservation.
